# Radiation-Induced Fibrotic Tumor Microenvironment Regulates Anti-Tumor Immune Response

**DOI:** 10.3390/cancers13205232

**Published:** 2021-10-19

**Authors:** Jae-Kyung Nam, Ji-Hee Kim, Min-Sik Park, Eun Ho Kim, Joon Kim, Yoon-Jin Lee

**Affiliations:** 1 Division of Radiation Biomedical Research, Korea Institute of Radiologic and Medical Sciences, Seoul 01812, Korea; jaek100@kirams.re.kr (J.-K.N.); wlgml9054@kirams.re.kr (J.-H.K.); bbrr0044@kirams.re.kr (M.-S.P.); 2 Laboratory of Biochemistry, Division of Life Sciences, Korea University, Seoul 02841, Korea; 3 Department of Biochemistry, School of Medicine, Daegu Catholic University, Nam-gu, Daegu 42472, Korea; eh140149@cu.ac.kr

**Keywords:** high linear energy transfer, programmed death-ligand 1, X-ray radiation therapy, neutron radiation therapy, anti-tumor immune response, fibrotic tumor microenvironment

## Abstract

**Simple Summary:**

Radiation therapy can modulate anti-tumor immune responses. In this study, we investigated the relationship between the anti-tumor immune response and tumor fibrosis after X-ray or neutron radiation therapy. Neutron radiation therapy resulted in lesser fibrosis and greater anti-tumor immunity compared to X-ray irradiation. Radiation therapy-induced fibrotic changes within the tumor environment and tumor regrowth were suppressed by specifically deleting Trp53 in endothelial cells. In particular, the upregulation of PD-L1 expression after X-ray radiation therapy was significantly suppressed via EC-Trp53 deletion. Understanding the effects of different radiation therapy types on the tumor microenvironment provides strategies for enhancing the efficacy of combined radio- and immunotherapy.

**Abstract:**

High linear energy transfer (LET) radiation, such as neutron radiation, is considered more effective for the treatment of cancer than low LET radiation, such as X-rays. We previously reported that X-ray irradiation induced endothelial-to-mesenchymal transition (EndMT) and profibrotic changes, which contributed to the radioresistance of tumors. However, this effect was attenuated in tumors of endothelial-specific Trp53-knockout mice. Herein, we report that compared to X-ray irradiation, neutron radiation therapy reduced collagen deposition and suppressed EndMT in tumors. In addition to the fewer fibrotic changes, more cluster of differentiation (CD8)-positive cytotoxic T cells were observed in neutron-irradiated regrowing tumors than in X-ray-irradiated tumors. Furthermore, lower programmed death-ligand 1 (PD-L1) expression was noted in the former. Endothelial-specific Trp53 deletion suppressed fibrotic changes within the tumor environment following both X-ray and neutron radiation therapy. In particular, the upregulation in PD-L1 expression after X-ray radiation therapy was significantly dampened. Our findings suggest that compared to low LET radiation therapy, high LET radiation therapy can efficiently suppress profibrotic changes and enhance the anti-tumor immune response, resulting in delayed tumor regrowth.

## 1. Introduction

Radiation therapy with high linear energy transfer (LET) radiation (alpha particles, protons, or neutrons) is more effective at killing tumor cells than low LET radiation, such as X-rays and γ-rays [1,2,3]. Unlike low LET radiation, which relies on the generation of reactive oxygen species for cytotoxicity, neutron radiation therapy does not depend on the presence of oxygen, and cellular damage is primarily mediated via nuclear interactions. However, the differences between the effects of low LET and high LET within the tumor microenvironment (TME) remain unclear [4,5].

Radiation therapy modulates the anti-tumor immune response [6,7]. Combinations of immune checkpoint blockade via antibodies against programmed death 1 (PD-1) and programmed death-ligand 1 (PD-L1) with radiotherapy have been explored in the clinic [8]. In fact, these were reported to be more effective than monotherapy [9]. Cancer-associated fibroblasts (CAFs), a major cell type within the TME, play an important role in tumorigenesis and tumor progression. Recent studies have reported that CAFs can control tumor immune escape and anti-tumor immune responses [10].

In response to low LET radiation, radio-resistant cancer cells are known to acquire endothelial-to-mesenchymal transition (ENDMT) phenotypes, thus giving rise to CAFs. Various target pathways, such as TGF-β, Wnt, and Notch, have been reported to drive ENDMT-mediated radioresistance [11]. Overall, it has been established that the EndMT promotes CAF formation in tumors [12]. Claire et al. reported that anti-PD-1 treatment prolonged survival when administered in parallel to radiation and TGF-β blockade [13].

In our previous study, the EndMT and fibrotic changes that occurred during tumor regrowth after radiation therapy (X-ray) were suppressed by endothelial cell-specific p53 deletion, which enhanced the efficacy of radiotherapy [14]. However, changes within the TME following radiation therapy with high LET have rarely been reported.

Various clinical trials on CAF-targeting therapy are currently ongoing [15]. Hyaluronidase reshapes the hyaluronic-rich stroma, facilitating CD8^+^ T-cell infiltration, and thus inhibiting tumor growth [16]. CAFs regulate extracellular matrix (ECM)-mediated T-cell trapping as a phenomenon of immune-exclusion patterns [15]. Moreover, CAFs have been reported to express PD-L1 and PD-L2, the expression levels of which are upregulated by TLR4 or IFN-γ, thereby promoting tumor immune escape [17]. TGF-β within the TME stimulates regulatory T cells (Tregs) and inhibits cytotoxic T cells [18]. Pirfenidone, an FDA-approved drug for idiopathic pulmonary fibrosis, inhibits the production of TGFb1 and collagen, reduces tumor growth, and increases T-cell and NK-cell infiltration in murine non-small-cell lung cancer (NSCLC) [19].

Herein, we studied the relationship between the anti-tumor immune response and TME fibrosis after X-ray (low LET) and neutron radiation (high LET) therapy. To this end, we analyzed the TME of regrowing tumors after radiation therapy, utilizing EC-Trp53- and Tgfbr2-knockout mice. Furthermore, we investigated the effects of 2-methoxyestradiol (2-ME), a putative EndMT inhibitor based on our previous report, on fibrosis and anti-tumor immunity. High LET therapy suppressed fibrosis and the ENDMT to a greater extent than low ENDMT, leading to lower tumor regrowth.

## 2. Materials and Methods

### 2.1. Mice

All animal experiments were performed according to the ARRIVE (Animal Research: Reporting of In Vivo Experiments) guidelines [20], with the approval of the Institutional Animal Care and Use Committee of the Korea Institute of Radiological & Medical Sciences.

The loxp-cre system was used to produce transgenic mice by crossing individuals homozygous for the target gene with those homozygous for the loxP flank and mice hemizygous for the Cre transgene. The desired transgenic mice were produced by crossing loxp homozygous for the *p53* or *Tgfbr2* gene with Cre recombinase at the *Tie2* promoter region that is specifically expressed in endothelial cells. Specific pathogen-free *Tie2*-Cre, *Trp53^flox/flox^*, and *Tgfbr2^flox/flox^* mice of a C57BL/6 background were purchased from the Jackson Laboratory. *Tie2*-Cre;*Trp53^flox/flox^* mice were obtained by crossing male *Tie2*-Cre mice with second-generation female *Trp53^flox/flox^* mice. *Tie2*-Cre;*Tgfbr2^+/flox^* mice were obtained by crossing first-generation male *Tie2*-Cre mice with female *Tgfbr2^flox/flox^* mice. *Tie2*-Cre^–/–^ littermates were used as controls. C57BL/6 mice that were used as tumor models for 2-ME treatment were purchased from Orient Bio. All experiments were conducted using 6–8-week-old mice.

### 2.2. Syngeneic Tumor Models

Tumor cells (KP cells) were previously isolated from the lung adenocarcinoma tumors of LSL-*Kras^G12D^*;*Trp53^flox/flox^* mice [21]. Tumor cells (5 × 10^5^) were subcutaneously injected into the right thigh of *Tie2*-Cre;*Trp53^flox/flox^* and *Tie2*-Cre;*Tgfbr2^+/flox^* mice. *Tie2*-Cre^–/–^ littermates were used as controls. The tumor (150–200 mm^3^) was irradiated using the X-RAD 320 platform (Precision X-ray, North Branford, CT) or fast neutron (MC-50; Scanditronix, Uppsala, Sweden). The tumor length (*L*), width (*W*), and height (*H*) were measured using calipers, and the tumor volume was calculated using the following formula: tumor volume = (*L* × *W* × *H*)/2. Fibrosis inhibitor 2-ME (Selleckchem, Houston, TX, USA; #S1233) was dissolved in DMSO and diluted in 30% PEG-400 with 1% Tween 80 solution. KP tumor-injected C57BL/6 mice were treated with 2-ME (60 mg/kg, intraperitoneally) 1 h before irradiation for a total of six times over 2 weeks.

### 2.3. Irradiation

A single dose of X-ray radiation (20 Gy) was delivered using the X-RAD 320 platform (Precision X-rays). Fast neutrons (20 GyE, 9.8 MeV, 30–40 keV/µm) were generated through the bombardment of protons with beryllium in a nuclear reaction within the MC-50 cyclotron (Scanditronix, Uppsala, Sweden). The absorbed dose and dose distribution of fast neutrons or X-rays were measured using a paired ionization chamber [22]. The gray equivalent (GyE) unit for neutron irradiation states the equivalent biological dose compared to X-ray therapy [23,24]. A neutron dose of 2.2 was calculated to have an equivalent cell-killing efficacy as X-rays, as determined via in vitro clonogenic assays based on relative biological effectiveness (RBE) [25]. 

### 2.4. Immunofluorescence and Immunohistochemistry

Tissues were fixed in 10% (v/v) neutral-buffered formalin, embedded in paraffin (FFPE), and sectioned. The sections were then deparaffinized and stained [26]. Sections were treated with antigen retrieval citrate buffer (Sigma, Burlington, MA, USA; #C9999) at 95 °C for 30 min and then in 0.3% H_2_O_2_ for 15 min. The cells were then permeabilized with PBS containing 0.1% Triton X-100 (PBST) for 15 min and blocked for 30 min with PBST containing normal horse serum. Immunofluorescence staining was performed in the same manner as immunohistochemistry, except for the 0.3% H_2_O_2_ step. We used primary antibodies against CD31 (endothelial cell marker; 1:200; R&D Systems, Minneapolis, MN, USA; #AF3628), αSMA (fibroblast marker; 1:1000; Sigma-Aldrich; #A5228), F4/80 (macrophage marker; 1:200; Abcam, Waltham, MA, USA; #ab6640), CD8 (cytotoxic T-cell marker; 1:200; Abcam; #ab22378), Granzyme B (activated cytotoxic T-cell marker; 1:200; Abcam; #ab4059), PD-1 (1:200; Abcam; #214421), PD-L1 (1:200; Abcam, #233482), and γH2AX (DNA damage marker; 1:200; Millipore, Rte Industrielle de la Hardt, Molsheim, FRA; #05–636). For immunohistochemistry, an ABC kit (Vector, Burlingame, CA, USA; #PK-6100) and DAB kit (Vector; #PK-4100) were used for staining, and the nuclei were stained with hematoxylin. For immunofluorescence, a secondary fluorescent antibody was used, and the nuclei were stained with DAPI (Sigma; #D9542).

Collagen deposition was assessed using Masson’s trichrome stain (Polyscience, Warrington, PA, USA; #25088-100). For quantification, a minimum of five images per tissue section were acquired, and positively stained areas were evaluated using ImageJ software (http://imagej.net/, accessed on 29 July 2021).

### 2.5. Statistical Analysis

The data are expressed as the mean ± standard deviation (SD) or mean ± standard error of the mean (SEM). One-way analysis of variance (ANOVA) with Tukey’s multiple comparisons test was used for multiple comparisons (for all others) in GraphPad Prism version 7.0. A *p*-value < 0.05 was considered to indicate statistical significance. The experimenters were blinded to group assignments and outcome assessment. We used Image J 1.49v (NIH), R 4.0.4, and Zen 3.2 (Zeiss) for data and image analyses.

## 3. Results

### 3.1. Neutron Radiation Therapy Efficiently Inhibits Tumor Regrowth and Fibrotic Changes under EC-Trp53 Knockout Conditions

**Figure 1:**
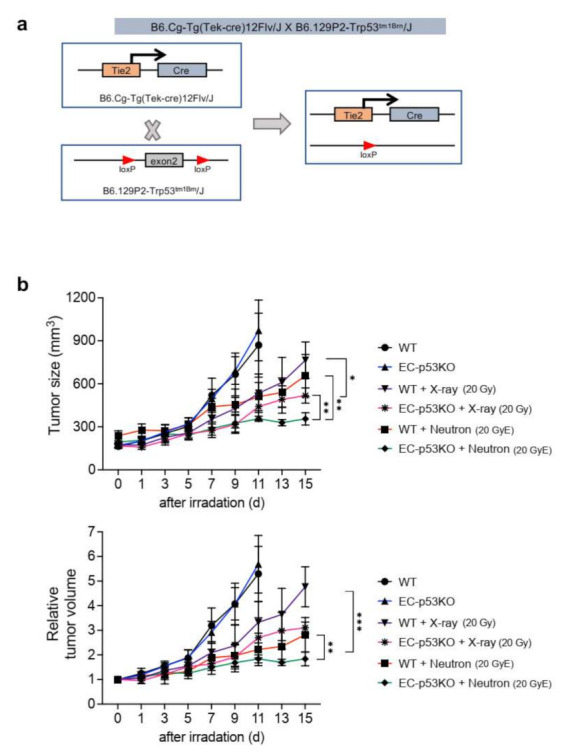
Endothelial-specific *Trp53* deletion inhibits KP tumor regrowth after neutron irradiation or X-ray irradiation. (**a**) Schematic diagram of the endothelial-specific *Trp53* exon2 deletion. *Trp53^flox/flox^* (WT) and *Tie2*-Cre;*Trp53^flox/flox^* (EC-p53KO) mice were subcutaneously injected with KP cells in the right thigh. (**b**) Tumor growth in WT and EC-p53KO mice after no irradiation, X-ray irradiation (20 Gy), and neutron irradiation (20 GyE). Irradiation was performed when tumor tissues reached a volume of 150–200 mm^3^. Non-irradiated tumor tissues were obtained on days 0 and 11, and X-ray- or neutron-irradiated tumor tissues were obtained on day 15 after irradiation (*n* = 6–7 animals per group). For (**b**), error bars indicate the standard deviation (SD); two-way analysis of variance (ANOVA) was used for multiple comparisons. ^*^ *p* < 0.05, ^**^ *p* < 0.01, and ^***^ *p* < 0.001.

**Figure 2:**
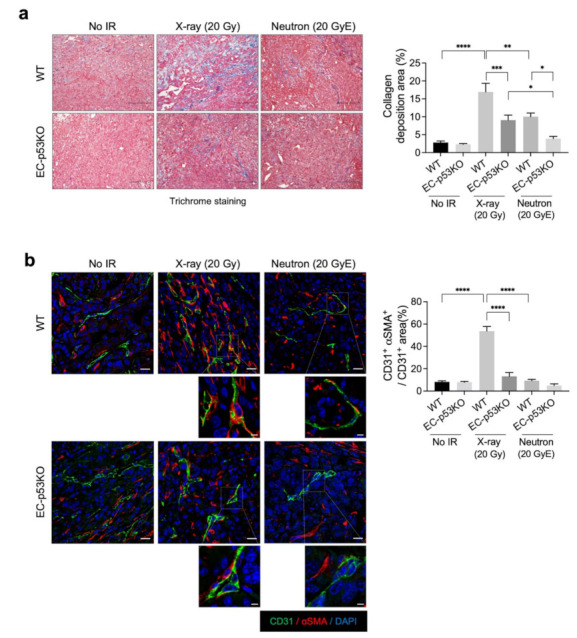
Lower collagen deposition is observed after neutron irradiation compared to X-ray irradiation, in addition to ENDMT suppression, in both WT and EC-p53KO mouse tumor models. Non-irradiated tumor tissues obtained on day 0 and tumor tissues obtained on day 15 after irradiation were used for staining. (**a**) Trichrome staining of irradiated or non-irradiated KP tumors from WT and EC-p53KO mice. Scale bar = 40 µm. Quantification of collagen deposition area per field as an average of five fields (magnification, 200×, n > 5). (**b**) Immunofluorescence staining of CD31 (green) and αSMA (red) expression in irradiated or non-irradiated KP tumors from WT and EC-p53KO mice. Scale bar = 20 μm; scale bar of cropped images = 5 μm. Quantification of the αSMA+CD31+ colocalization area relative to CD31+ area as an average of 5 fields (magnification, 200×, *n* > 5). For all graphs, error bars indicate the SEM (one-way ANOVA for multiple comparisons). * *p* < 0.05, ** *p* < 0.01, *** *p* < 0.001 and **** *p* < 0.0001.

We previously reported that X-ray radiation-induced EndMT enhanced vascular fibrosis and collagen deposition, especially around tumor vessels within the TME. Further, endothelial Trp53 deletion inhibited the radiation-induced EndMT and subsequent fibrotic changes within the TME, as observed in an EC-specific Trp53-knockout (EC-p53KO) mouse model [14]. To explore the effects of neutron irradiation on the TME, we examined tumor regrowth, collagen deposition, and EndMT after radiation therapy in EC-p53KO mice (Figure 1 and Figure 2). We generated a syngeneic tumor model using tumor cells (referred to as KP cells) from a spontaneous NSCLC tumor isolated from conditional *Kras^G12D^*;*Trp53^flox/flox^* mice (Figure 1a) [21]. 

Tumor growth in WT mice was delayed after treatment with 20 GyE neutron irradiation, compared to that after 20 Gy X-ray irradiation; however, there was no statistically significant difference in tumor growth between the two irradiation groups. EC-p53KO mice exhibited significantly delayed tumor growth compared to their WT counterparts after both X-ray and neutron radiation therapy (Figure 1b). 

Increased collagen deposition was observed in WT tumors after X-ray irradiation, which was significantly attenuated in EC-p53KO tumors (Figure 2a). Neutron irradiation significantly decreased collagen deposition in WT tumors compared to X-ray-irradiated WT tumors, with an even greater decrease observed in EC-p53KO tumors (Figure 2a). In parallel to collagen composition, the EndMT, indicated by the co-localization of CD31 (EC marker) and αSMA (fibroblastic marker), was significantly suppressed after neutron irradiation compared to X-ray irradiation in WT tumors (Figure 2b).

The results indicate that neutron radiation therapy causes fewer fibrotic changes compared to X-ray radiation therapy, as indicated by collagen deposition and EndMT occurrence within the irradiated TME. Consequently, lower tumor regrowth was observed.

### 3.2. Neutron Radiation Therapy Enhanced the Tumor Infiltration of CD8^+^ Cytotoxic T Cells 

We previously reported that the radiation-induced EndMT can promote the polarization of tumor-associated macrophages and the tumor infiltration of cytotoxic T cells [14]. Herein, we hypothesized that low LET and high LET would have distinct effects on tumor immunity.

**Figure 3:**
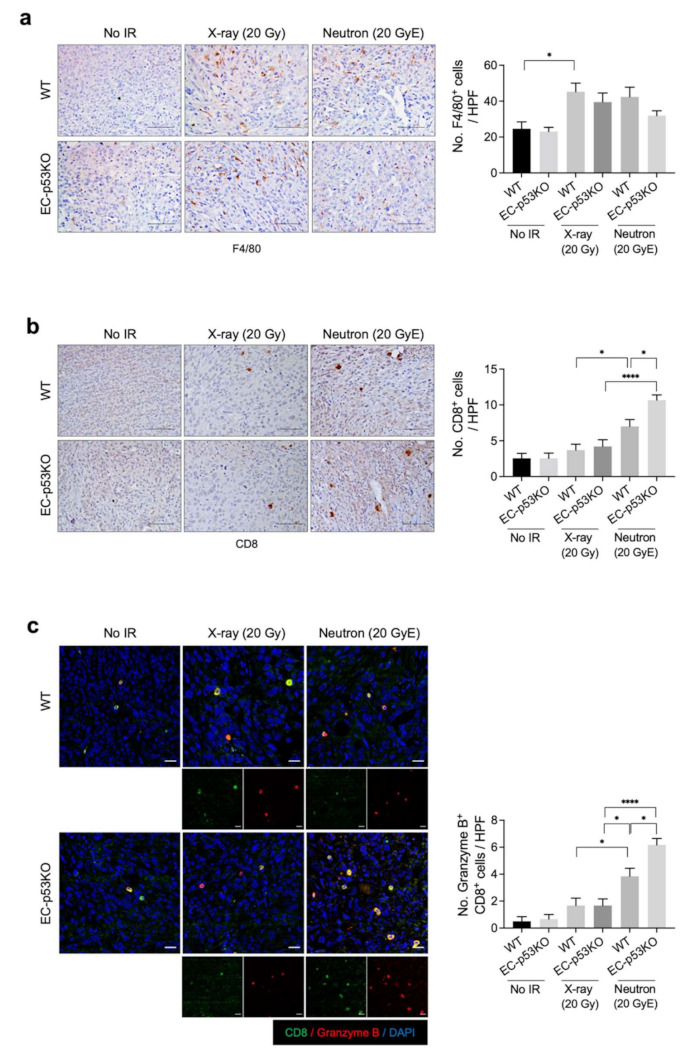
Neutron irradiation upregulates the infiltration of cytotoxic T cells to a greater extent compared to X-ray irradiation in both WT mice and EC-p53KO mice. Non-irradiated tumor tissues obtained on day 0 and tumor tissues obtained on day 15 after irradiation were subjected to staining. (**a**) Immunohistochemistry of F4/80 (brown color) in KP tumors from WT and EC-p53KO mice, irradiated or non-irradiated. Scale bar = 40 µm. Quantification of F4/80+ (macrophages) cells per field as an average of five fields (magnification, 200×, *n* > 5). (**b**) Immunohistochemistry of CD8+ (brown color) in irradiated and non-irradiated KP tumors from WT and EC-p53KO mice. Scale bar = 40 µm. uantification of an average of five fields with a prominent CD8+ cell population (cytotoxic T cells) per field (magnification, 200×, *n* > 5). (**c**) Immunofluorescence staining of CD8+ (green) and granzyme B+ (red) cells in irradiated or non-irradiated KP tumors from WT and EC-p53KO mice. Scale bar = 20 µm. Quantification of an average of five fields with a prominent CD8+ granzyme B+ (active cytotoxic T cells) cell population per field. (magnification: 200×; *n* > 5). For all graphs, error bars indicate the SEM (one-way ANOVA for multiple comparisons). * *p* < 0.01 and **** *p* < 0.0001.

To assess this, we compared the effects of neutron and X-ray irradiation on immune cell composition within the TME. Immunohistochemical analysis revealed that the population of F4/80+ macrophages within irradiated tumors of WT mice increased significantly after both X-ray and neutron irradiation compared to their populations in non-irradiated WT tumors. No such significant difference was observed in the tumors of EC-p53KO mice (Figure 3a). Granzyme B^+^CD8^+^ cytotoxic T cells were significantly increased following neutron irradiation compared to X-ray irradiation in WT tumor models. This effect was further enhanced in EC-p53KO mice (Figure 3b,c).

These results suggest that neutron radiation therapy combined with ENDMT inhibition could enhance anti-tumor immunity by increasing cytotoxic T-cell infiltration into tumors. Moreover, we suggest that the population of CD8^+^ cytotoxic T cells increased in neutron radiation therapy, which was further enhanced by EC-p53 deletion, leading to fibrotic changes in the TME.

### 3.3. PD-L1 Expression Is Upregulated after X-ray Irradiation but Not after Neutron Irradiation 

**Figure 4:**
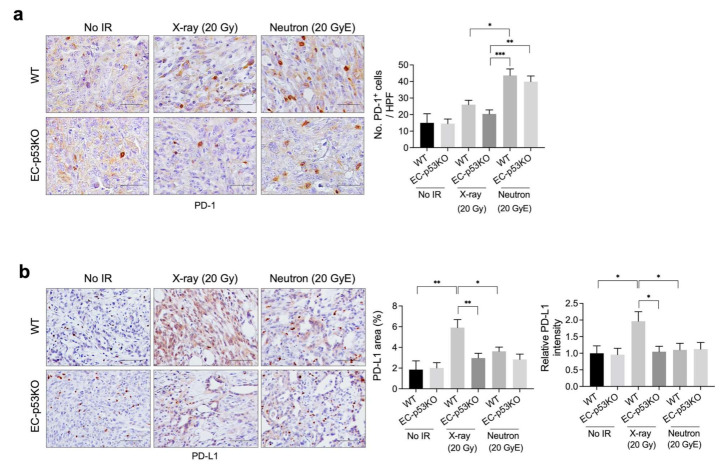
Neutron irradiation induces lower PDL-1 expression than X-ray irradiation in the KP tumors of WT mice and EC-p53KO mice. Non-irradiated tumor tissues obtained on day 0 and tumor tissues obtained on day 15 after irradiation were used for staining. (**a**,**b**) Immunohistochemistry of PD-1 (brown color) and PD-L1 (brown color) in irradiated and non-irradiated KP tumors from WT and EC-p53KO mice. (**a**) Scale bar = 20 µm; (**b**) Scale bar = 40 µm. Quantification of an average of five fields with a prominent PD-1+ cell population and a high PD-L1-positive area per field (magnification, 200×, *n* > 5). For all graphs, error bars indicate the SEM (one-way ANOVA for multiple comparisons). * *p* < 0.05, ** *p* < 0.01, *** *p* < 0.001.

As radiation is known to regulate immunity within the TME, in part by enhancing antigen presentation and PD-L1 expression [27], we examined tumor PD-L1 expression after X-ray and neutron irradiation. Immunohistochemical analysis revealed that the population of PD-1^+^ immune cells was higher in neuron-irradiated tumors than in X-ray-irradiated tumors, whereas no significant difference was observed in EC-p53KO tumors (Figure 4a). Furthermore, PD-L1 expression was lower in neutron-irradiated tumors than in X-ray irradiated tumors. EC-p53-KO tumors exhibited lower PD-L1 expression compared to WT following both X-ray and neutron irradiation (Figure 4b).

Thus, tumor PD-L1 expression was upregulated following X-ray radiation therapy and neutron radiation therapy compared to that in non-irradiated tumors, suggesting that anti-PD-L1 immune therapy may be effective in combination with X-ray or neutron irradiation for the treatment of NSCLC.

### 3.4. PD-L1 Expression Was Upregulated by EC-Tgfbr2 Knockdown, with Increased Fibrosis Observed after X-ray Irradiation

**Figure 5:**
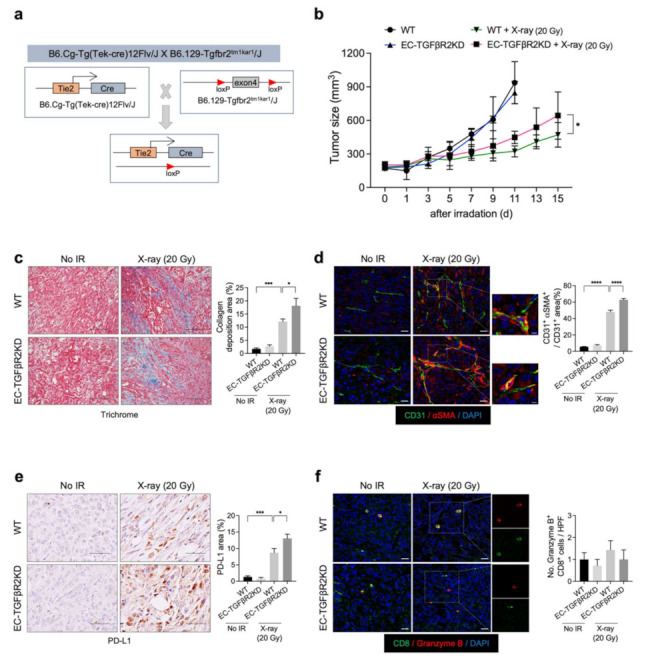
Endothelial-specific *TGFβR2* knockdown enhances KP tumor regrowth, collagen deposition, EndMT, and PD-L1 expression, but not cytotoxic T-cell infiltration, after X-ray irradiation. (**a**) Schematic diagram of the endothelial-specific *TGFβR2* exon4 knockdown. *Tgfbr^+/flox^* (WT) and *Tie2*-Cre; *Tgfbr2^+/flox^* (EC-TGFβR2KD) mice were subcutaneously injected with KP cells in the right thigh. Non-irradiated tumor tissues obtained on day 0 and irradiated tumor tissues obtained on day 15 after irradiation were used for staining. (**b**) WT and EC-TGFβR2KD tumor growth in irradiated and non-irradiated mice. Irradiation was performed when tumor tissues reached 150–200 mm^3^. Non-irradiated tumor tissues were obtained on days 0 and 11 after irradiation, and irradiated tumor tissues were obtained on day 15 after irradiation (*n* = 5–6 animals per group). Error bars indicate the SD (two-way ANOVA for multiple comparison). (**c**) Trichrome staining of KP tumors from irradiated or non-irradiated WT and EC-TGFβR2KD mice. Scale bar = 20 µm. Quantification of collagen deposition area per field as an average of five fields (magnification, 200×, *n* > 5). (**d**) Immunofluorescence staining of CD31 (green) and αSMA (red) expression in irradiated or non-irradiated KP tumors from WT and EC-TGFβR2KD mice. Scale bar = 20 μm; scale bar of cropped images = 5 µm. Quantification of the αSMA^+^CD31^+^ colocalization area per CD31^+^ area as an average of five fields (magnification, 200×, *n* > 5). (**e**) Immunohistochemistry of PD-L1 (brown color) in irradiated or non-irradiated KP tumors from WT and EC-TGFβR2KD mice. Scale bar = 20 µm. Quantification of PD-L1 area per field as an average of five fields (magnification, 200×, *n* > 5). (**f**) Immunofluorescence staining of CD8 (green) and granzyme B (red) cells in irradiated and non-irradiated KP tumors from WT and EC-specific TGFβR2KD mice. Scale bar = 20 µm. Quantification of an average of five fields with a prominent CD8^+^ granzyme B^+^ cell population per field (magnification, 200×, *n* > 5). For graphs (c–f), error bars indicate the SEM (one-way ANOVA for multiple comparisons). ^*^ *p* < 0.05, ^***^ *p* < 0.001, and ^****^ *p* < 0.0001.

TGF-β signaling is important in ENDMT regulation and the tumor response to radiation [28,29,30]. Previously, we reported that EC-Tgfbr2 knockdown promotes the tumor EndMT and subsequent collagen deposition within TME, especially around tumor vessels, thus promoting tumor growth [14]. To examine the potential relationship between tumor radiation-induced collagen deposition and PD-L1 expression, we generated EC-specific Tgfbr2 knockdown (EC-TGFβR2KD) mice (*Tie2*-Cre;*Tgfbr2^flox/+^*) (Figure 5a). Enhanced tumor regrowth was observed after X-ray irradiation in the Tgfbr2 knockdown mice compared to the WT mice, in agreement with the patterns of collagen deposition and EndMT (Figure 5b–d). Furthermore, the radiation-induced upregulation of PD-L1 was more prominent in Tgfbr2 knockdown mice than in WT mice (Figure 5e). However, no significant increase in activated cytotoxic T cells was observed (Figure 5f). These results suggest that PD-L1 upregulation after radiation therapy correlates with TME fibrosis after radiation therapy.

### 3.5. 2-ME Reduces Radiation-Induced PD-L1 Expression

We previously reported that 2-ME inhibits radiation-induced fibrosis and EndMT in spontaneous lung adenocarcinoma mouse models [31]. To further explore the potential relationship between TME fibrosis and PD-L1 upregulation, we examined the effects of 2-ME on radiation-induced PD-L1 expression in syngeneic KP tumor mouse models. 

**Figure 6:**
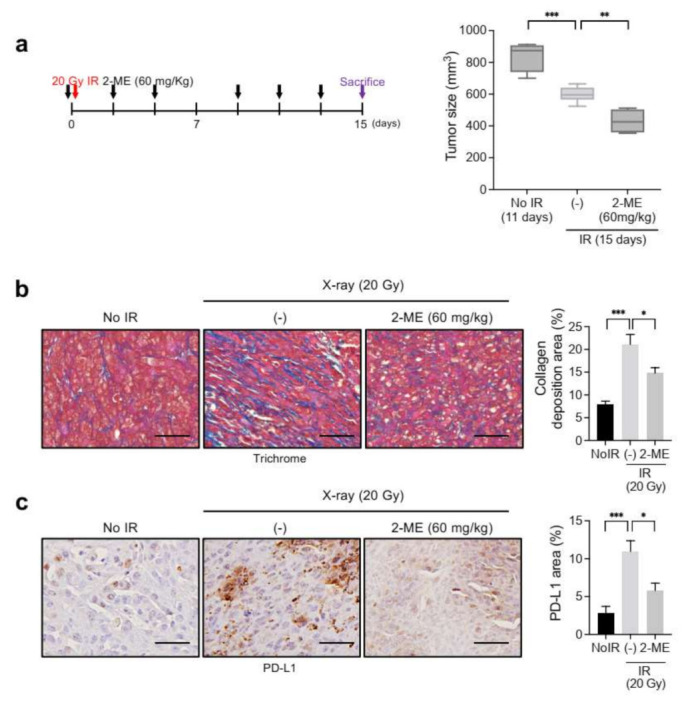
2-ME inhibits KP tumor regrowth, collagen deposition, and PD-L1 expression after X-ray irradiation. C57BL/6 mice were subcutaneously injected with KP cells in the right thigh. Irradiation and 2-ME (60 mg/kg) injections were performed when tumor tissues reached a volume of 150–200 mm^3^. Mice were intraperitoneally (i.p.) injected with 2-ME 1 h before irradiation for a total of six times over 2 weeks. Non-irradiated tumor tissues obtained on day 0 and irradiated tumor tissues obtained on day 15 were used for staining. (**a**) KP tumor growth with or without X-ray irradiation and with or without 2-ME (*n* = 3–6 animals per group). Error bars indicate the SD (two-way ANOVA for multiple comparisons). (**b**) Trichrome staining of collagen deposition in irradiated or non-irradiated KP tumors, with or without 2-ME. Scale bar = 20 µm. Quantification of collagen deposition area per field as an average of five fields (magnification, 200×, *n* > 5). (**c**) Immunohistochemistry of PD-L1 (brown color) in irradiated or non-irradiated KP tumors, with or without 2-ME. Scale bar = 20 µm. Quantification of PD-L1 area per field as an average of five fields (magnification, 200×, *n* > 5). For graphs (c–f), error bars indicate the SEM (one-way ANOVA for multiple comparisons). ^*^ *p* < 0.05, ^**^ *p* < 0.01, and ^***^ *p* < 0.001.

2-ME (60 mg/kg) was intraperitoneally administered 1 h before X-ray irradiation for a total of six times over 2 weeks. 2-ME treatment significantly inhibited tumor size at 15 days after irradiation (Figure 6a). Trichrome staining revealed that collagen deposition was reduced in irradiated tumors with 2-ME treatment compared to irradiated tumors without it (Figure 6b). Consistently, the expression of PD-L1 was also reduced in 2ME-treated irradiated tumors (Figure 6c). These results suggest that the expression of PD-L1 depends, in part, on the extent of collagen deposition during tumor regrowth after X-ray radiation therapy.

## 4. Discussion

The current findings support the differential effects of neutron and X-ray radiation therapy on the TME in lung adenocarcinoma syngeneic tumor models. Neutron radiation therapy delayed tumor regrowth, inducing lower collagen deposition, greater cytotoxic T-cell infiltration, and lower PD-L1 expression compared to X-ray irradiation. Based on these findings, we concluded that high LET treatment had more favorable effects on TME fibrosis and anti-tumor immunity. Nevertheless, a limitation of the current study was that the anti-tumor immune response in irradiated tumors was evaluated only based on immune cell composition. We are currently carrying out a study on anti-PD-L1/PD-1 in combination with high LET/low LET radiation therapy.

Several previous works have suggested that high and low LET irradiation produce distinct effects [32,33,34]. However, the specific molecular changes within the TME following high and low LET radiation therapy have remained unclear. In the present study, we confirmed the differential effects of high and low LET radiation therapy on the EndMT and subsequent collagen deposition within the TME. Future studies on changes in EndMT-related gene expression are required to validate the present findings. 

Radiation therapy is known to immunologically regulate the TME, in part through changes in PD-L1 expression [35]. It was recently reported that anti-PD-L1 antibody treatment following radiation therapy improved progression-free survival in NSCLC patients [36].

In the present study, EC-p53 deletion inhibited X-ray radiation-induced EndMT and subsequent tumor fibrosis, as indicated by the greater infiltration of cytotoxic T cells and the lower PD-L1 expression. In contrast, enhanced fibrosis and PD-L1 expression were observed in EC-TGFR2 knockdown tumors after X-ray irradiation. Furthermore, 2-ME, an EndMT inhibitor, can effectively inhibit X-ray radiation-induced PD-L1 expression. Thus, we suggest that regulating the radiation-induced EndMT and subsequent fibrosis can affect the infiltration of cytotoxic T cells and PD-L1 expression in tumors. It has been reported that collagen upregulation correlates with PD-l/PD-L1 blockade resistance in lung tumors, in parallel to a reduction in infiltrating cytotoxic T cells. Furthermore, TGF-β signaling- and ECM-related gene expression, including that of collagen, have been reported to correlate with PD-1/PD-L1 resistance [37]. We are currently exploring whether 2-ME treatment and EC-Tgfbr2 knockdown affect tumor regrowth following neutron radiation therapy. In addition, we suggest that the effectiveness of strategies targeting TME fibrosis may increase when combined with X-ray radiation therapy, as the latter promotes TME fibrosis.

Compared to X-ray irradiation, neutron radiation therapy leads to a lesser upregulation in PD-L1 expression and an increase in the population of PD-1^+^ and cytotoxic T cells. Since PD-1 plays a major role in CD8^+^ T-cell exhaustion, anti-PD-1 therapy can enhance anti-immune response in neutron therapy. Moreover, anti-PD-L1/PD-1 therapy may exert its effect systemically via PD-L1-overexpressing circulating mononuclear cells or lymphocytes in neutron radiation therapy. The present elucidated the effects of immune therapy combined with low LET and high LET radiation therapy. 

## 5. Conclusions

The present study demonstrated that neutron irradiation induces fewer fibrotic changes and lower PD-L1 expression within the TME than X-ray irradiation, in addition to a greater cytotoxic T-cell infiltration. Thus, the suppression of fibrosis and EndMT within the TME enhance anti-tumor immunity after radiation therapy. Understanding the differential immunological changes following high and low LET radiation therapy provides insights into enhancing the efficacy of radiation and immunotherapy combination regimens.

## Data Availability

All other relevant data are available from the corresponding author upon reasonable request.
